# The Prevalence of Fibromyalgia Among Medical Students at King Abdulaziz University: A Cross-Sectional Study

**DOI:** 10.7759/cureus.12670

**Published:** 2021-01-12

**Authors:** Abeer A Samman, Raneem A Bokhari, Sarah Idris, Rafal Bantan, Rahaf R Margushi, Sara Lary, Raghad M Sait, Yasser M Bawazir

**Affiliations:** 1 Internal Medicine, King Abdulaziz University Hospital, Jeddah, SAU; 2 Internal Medicine: Rheumatology, King Abdulaziz University Hospital, Jeddah, SAU; 3 Medicine, King Abdulaziz University Hospital, Jeddah, SAU; 4 Pediatrics, King Abdulaziz University Hospital, Jeddah, SAU

**Keywords:** fibromyalgia, medical students, prevalence, healthcare professions, stress

## Abstract

Background and objective

Fibromyalgia (FM) is a chronic, multifactorial pain condition. The latest literature suggests that genetic and environmental factors including continuous stress contribute significantly to FM's pathophysiology. In this study, we aimed to investigate the prevalence of FM among medical students as they are considered a population significantly at risk of developing the condition.

Methods

This cross-sectional study was conducted at King Abdulaziz University. Medical students included in the study were recruited through a random stratified sampling method. A self-administered questionnaire was distributed to the participants; it included questions related to widespread pain index (WPI) and symptom severity scale (SSS) to assess the symptoms and diagnosis of FM, which were established based on the current diagnostic criteria. All first-year students were excluded from this research.

Results

A total of 450 participants were recruited for the study. Among them, 291 (64.7%) were females and 159 (35.3%) were males. Their ages ranged from 18 to 26 years, and the mean age was 21.52 years (SD: ±1.52). They came from different academic levels: 97 (21.6%) were in the second year, 79 (17.6%) were in the third year, 70 (15.6%) were in the fourth year, 99 (22%) were in the fifth year, and 105 (23.3%) were in the sixth year. The overall prevalence of FM was found to be 43 (9.6%). It was established based on the number of students who fulfilled the diagnostic criteria or were previously diagnosed with FM by a professional physician.

Conclusion

FM is highly prevalent among medical students. Our findings demonstrate the likelihood of the influence of medical school on causing the condition, as it has a stressful education system with high academic expectations. We recommend that this issue be seriously addressed since FM leads to a significant burden on the students and can negatively affect their future medical practice.

## Introduction

Fibromyalgia (FM) is a chronic disease, and multiple factors contribute to its development; however, its main etiology is not entirely understood [1]. It is characterized by widespread musculoskeletal pain, usually described as a deep, throbbing, intense, and persistent ache in the muscles, accompanied by generalized burning and tingling [2,3]. The diagnosis is primarily based on chronic pain that is generalized with tenderness at the time of the physical exam, along with fatigue, sleep problems, notable cognitive dysfunction, mood changes, and headaches [4].

Recent studies have shown remarkable progress towards demonstrating the exact process involved in the development of chronic pain and other symptoms of FM [5]. Hence, the genetic predisposition related to FM and other disorders with chronic pain has been widely studied, and significant efforts have been made to understand the modifications occurring in the central nervous system in pain transmission and processing, which seems to greatly contribute to the clinical features of FM [6,7]. Pain in FM is not associated with tissue inflammation. Therefore, despite experiencing potentially disabling body pain, patients with FM do not develop tissue damage or deformity [8].

In conjunction with genetic backgrounds, several factors have been associated with the occurrence of FM; one factor that has been shown to play a significant role is stress, and numerous studies have focused on analyzing how different forms of stress can contribute to the development of FM [9].

The existing literature has shed light on the significantly high incidence of stress among students of health professions [10,11]. One study has reported that the highest incidence of stress is in fact found among medical students [12]. The current literature suggests that many students begin to experience a decline in their mental well-being once they start medical school, and it continues to be impaired throughout their training period due to their stressful lifestyle [13]. Medical students are a unique population as they face significantly varied types of stressors [14], such as peer competitiveness, the need to continuously memorize great amounts of information within a limited time, and constant evaluation. Additionally, they get very little time for enjoyment and relaxation and have to take up increased responsibilities associated with patient care [15]. All these factors make medical students vulnerable to increased risk of developing this type of chronic pain condition.

To our knowledge, this is the first study to be conducted in the region that addresses the prevalence of FM among Saudi medical students. Our primary aim in this study was to estimate the prevalence of FM among medical students of both basic and clinical years at King Abdulaziz University. We also aimed to investigate the association between FM and the varied demographics features among students.

## Materials and methods

This cross-sectional study was conducted at King Abdulaziz University Hospital, a tertiary healthcare center in Jeddah, Saudi Arabia, from February 2020 to April 2020. The study was approved by the Institutional Research Board of King Abdulaziz University Hospital.

A pre-designed electronic questionnaire based on Google Forms format was distributed to the medical students at different academic levels (from the second to sixth years) at King Abdulaziz University. Each student was provided detailed written information about the study and asked to give consent if they wished to participate in the study. All students who agreed to participate in the study were included. Students were invited to complete the Fibromyalgia Survey Diagnostic Criteria (FSDC), which had been validated in a previous cross-sectional study involving 1,665 FM patients [16]. It includes the American College of Rheumatology (ACR) 2010 diagnostic criteria for FM. Students who reported ever being told by a physician or healthcare provider that he or she had FM were also included in this study. First-year students were excluded, as according to the academic system at King Abdulaziz University, the first year is a preparatory year in which non-medical subjects are studied, and therefore our research specifications did not apply to them. Students with trauma or inflammatory rheumatological disease were also excluded. In addition, students in all other medical-field specialties and medical interns were excluded from this study.

The sample size required for this study was calculated to be 385, as it was determined to be the minimum number of participants needed for a 95% confidence level and a margin of error of 5 for this study. The calculations were made using the sample size calculation equation for cross-sectional studies [17]. However, the number of participants ultimately included in this study was 450, as more students expressed their willingness to participate in the study. We used a stratified random sampling technique. Firstly, the portion of students in each academic level was considered as a stratum; further, we randomly asked students of both male and female divisions to complete the questionnaire.

The study questionnaire included demographic characteristics, including age, gender, and academic level. In addition, students were further queried about their sleep hours per night for the past four weeks and about the presence of chronic comorbidities; they were also asked pain-related questions: whether they consumed any medications for pain or if their pain affected their ability to exercise. The questionnaire also asked whether their family or friends were causing any emotional stress to them due to their chronic pain condition.

FSDC consists of widespread pain index (WPI), which presents the number (a 0-19 count) of sites reported as being painful by the participant in the past week, and the symptom severity scale (SSS), which addresses the three main characteristics of FM: fatigue, waking unrefreshed, and cognitive symptoms over the past week. In addition, the extent of the somatic symptoms the participants were suffering from in general was also addressed (a final score of 0-12).

We defined FM by using the current diagnostic criteria of FM, which includes WPI, which is a scoring system that helps to localize the site of the pain, and SSS [18]. A response was considered positive when participants recorded a WPI score of ≥7/19 for pain sites and an SSS score of ≥5/12, or if they achieved a WPI score between 3 and 6/19 and an SSS score of ≥9/12; another criterion was symptoms being present at a similar level for at least three months, and the participants not having any another disorder that would otherwise explain the pain [18].

Statistical analysis was performed using the SPSS Statistics version 21 (IBM, Armonk, NY). Descriptive analysis using numbers and percentages were used to present demographics features and the prevalence of FM. Continuous variables were described as mean ±SD, and nonparametric distributions were presented as median ± interquartile range (IQR). A nonparametric χ2 (chi-squared) test was used to compare different categorical variables between participants with FM and those without FM. All statistical tests and confidence intervals were performed at α=0.05 (2-sided). Subsequently, a p-value of less than 0.05 was considered statistically significant.

## Results

A total of 450 participants were included in the analysis. Among them, 291 (64.7%) were females and 159 (35.3%) were males. Their age ranged from 18 to 26 years, with a mean age of 21.52 years (SD ±1.52). The participants came from different academic levels: 97 (21.6%) were in the second year, 79 (17.6%) were in the third year, 70 (15.6%) were in the fourth year, 99 (22%) were in the fifth year, and 105 (23.3%) were in the sixth year (Table 1).

**Table 1:** Characteristics of subjects by gender

Characteristic	Male (n=159)	Female (n=291)	Total (n=450)
Age in years, mean (SD)	21.79 (1.68)	21.37 (1.42)	21.52 (1.52)
Academic level			
Second-year, n (%)	41 (25.8%)	56 (19.2%)	97 (21.6%)
Third-year, n (%)	16 (10.1%)	63 (21.6%)	79 (17.6%)
Fourth-year, n (%)	26 (16.4%)	44 (15.1%)	70 (15.6%)
Fifth-year, n (%)	30 (18.9%)	69 (23.7%)	99 (22%)
Sixth-year, n (%)	46 (28.9%)	59 (20.3%)	105 (23.3%)

Among the participants, 43 (9.6%) had fulfilled the ACR diagnostic criteria for FM in that they had had it for a duration of at least three months. Participants who met the criteria were predominantly female [35 (81.4%)], and only eight (18.6%) were male.

**Figure 1:**
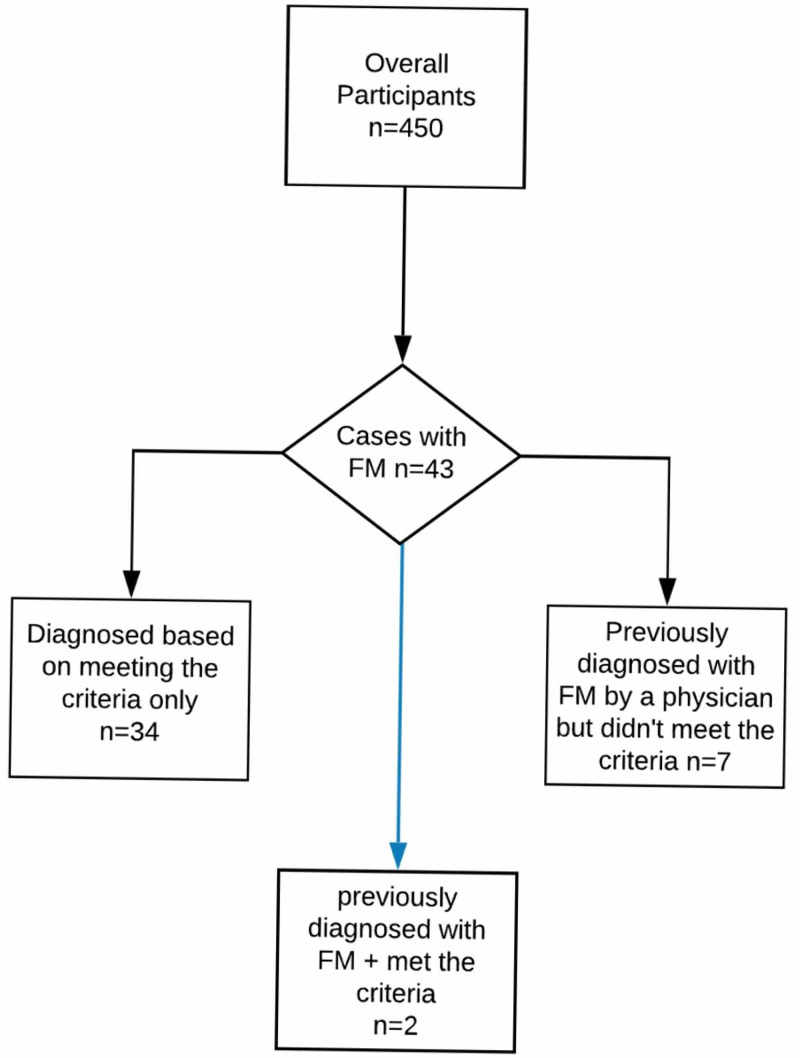
Participants with FM included in the study FM: fibromyalgia

Among the 43 cases who were considered to have FM, a minority of seven cases did not satisfy the ACR diagnostic criteria used in this study, but rather they reported they had received a previous FM diagnosis from a physician. At the same time, another two cases reported a previous diagnosis from a physician, but they also fulfilled the diagnostic criteria. Hence, there were a total of nine participants who reported that they had been diagnosed previously with FM by a physician. Accordingly, only 22.2% of the overall number of cases reported a physician’s diagnosis of FM along with meeting the diagnostic criteria (Figure 1).

In order to assess the effect of the academic level on the development of FM symptoms, we analyzed the prevalence of FM among different academic levels. Nine (20.9%) participants among the overall subjects with FM were in the second year, 11 (25.6%) were in the third year, five (11.6%) were in the fourth year, eight (18.6%) were in the fifth year, and 10 (23.3%) were in the 6th year. No significant difference was found between these groups (p=0.649).

**Figure 2:**
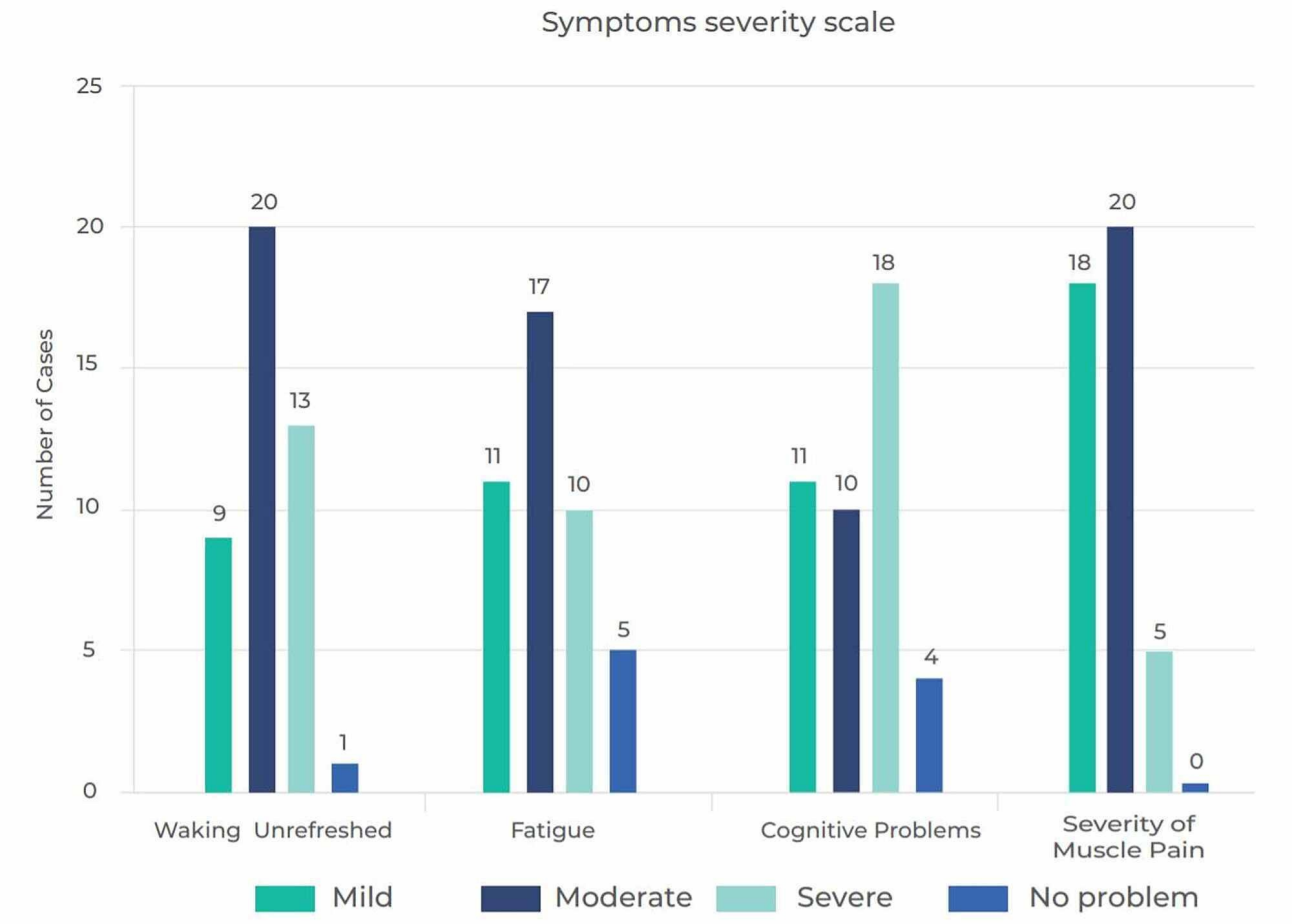
Symptom severity scale in subjects with FM FM: fibromyalgia

We also assessed the severity of FM symptoms: fatigue, waking up feeling unrefreshed and cognitive dysfunction targeting memory and concentration problems among the participants who met the criteria (Figure 2). All symptoms showed a significant statistical difference (p:<0.001). When evaluating the sleeping hours of the participants, 25 (58.1%) of those with FM and 237 (57.8%) of those without FM reported that they had slept less than seven hours per night in the past two weeks.

Other somatic symptoms were also assessed, and they included the following: irritable bowel syndrome, muscle pain, anxiety, depression, headache, heartburn, chest pain, and back pain (Table 2).

**Table 2:** Comparison between subjects with FM and those without FM based on gender and the presence of somatic symptoms P-values are based on the chi-squared test FM: fibromyalgia; IBS: irritable bowel syndrome

		Fibromyalgia, n (%)	No fibromyalgia, n (%)	P-value
Gender	Male	8 (18.6%)	151 (37.1%)	0.025
	Female	35 (81.4%)	256 (62.9%)	
Presence of somatic symptoms	Anxiety	36 (83.7%)	241 (59%)	0.003
	Depression	17.6 (79.1%)	150 (36.9%)	<0.001
	Muscle pain	35 (81.4%)	168 (41.3%)	<0.001
	IBS	24 (55.8%)	107 (26.3%)	<0.001
	Headache	34 (79.1%)	211 (51.8%)	0.001
	Heartburn	20 (46.5%)	88 (21.6%)	0.001
	Chest pain	16 (37.2%)	51 (12.5%)	<0.001
	Back pain	37 (86.0%)	171 (42.0%)	<0.001

Among our subjects with FM symptoms, 13 (32.6%) reported that they exercised regularly and 29 (67.4) reported they did not. When we asked whether their FM symptoms (pain specifically) affected their ability to exercise, 24 (55.8%) answered that pain did affect their exercise routine. Among the participants who did not meet the FM diagnostic criteria, 198 (48.6%) reported exercising regularly and 209 (51.4%) said they did not, and only 118 (29%) reported pain during exercise whereas 289 (71.0%) did not report any pain. This difference between participants with FM symptoms and those without FM regarding pain affecting their ability to exercise showed a statistically significant association (p=0.001).

**Figure 3:**
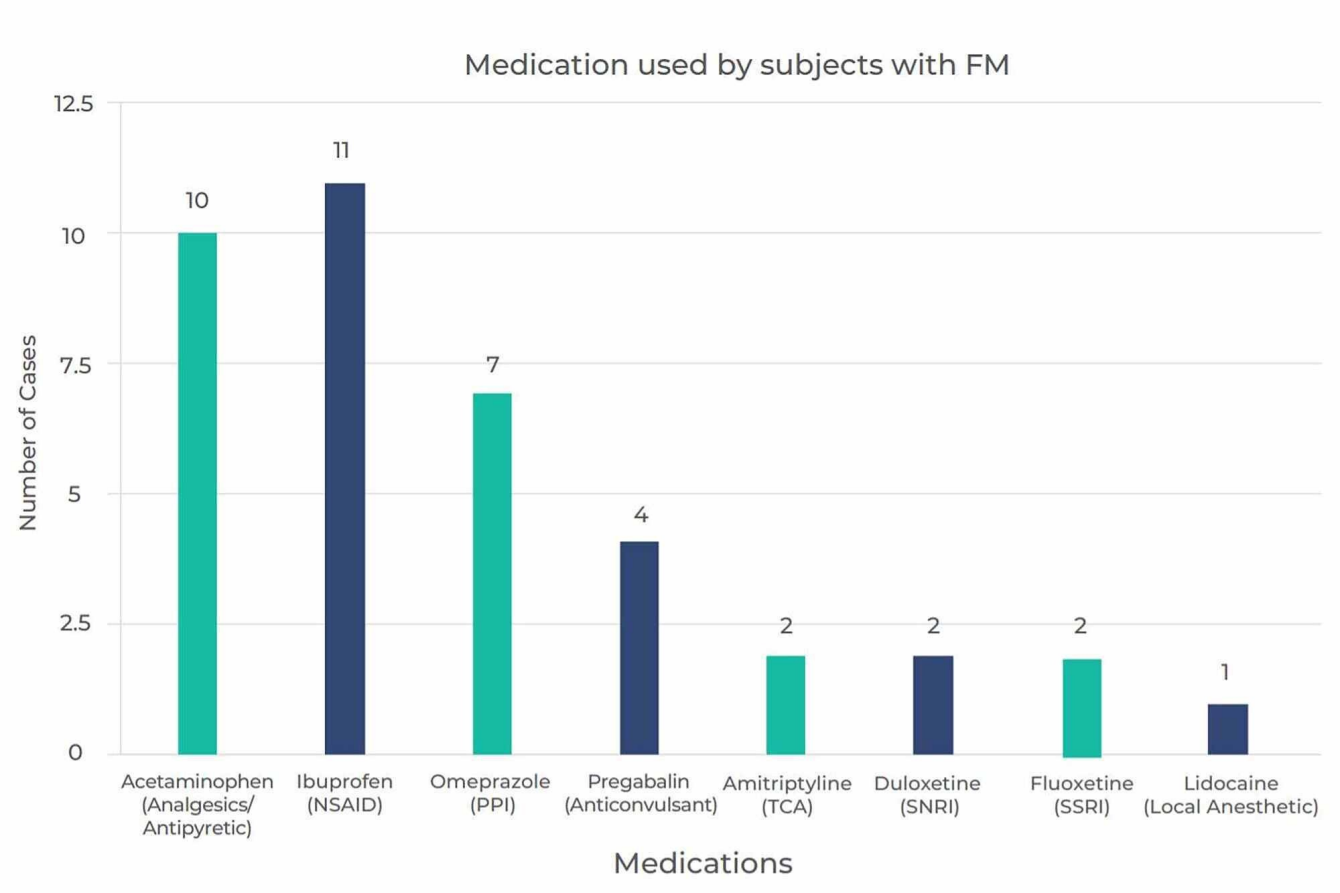
Medications used by participants with FM FM: fibromyalgia; NSAID: non-steroidal anti-inflammatory drug; PPI: proton pump inhibitor; TCA: tricyclic antidepressant; SNRI: selective serotonin and norepinephrine reuptake inhibitor; SSRI: selective serotonin reuptake inhibitor

When we asked the participants with FM whether they took any medications for their symptoms, 25 (58.1%) reported that they did not take any medication to control their symptoms, while 18 (41.9%) reported that they took some sort of medication to control their symptoms (Figure 3). Further investigation of the subjects with FM on medications revealed that their median WPI score was 7 (IQR: 4-12). Similarly, those who were not on medications also scored 7 points on their WPI (IQR: 5-9).

## Discussion

In this study, we aimed to evaluate the prevalence of FM among medical students, a population that is particularly vulnerable to overwhelming stress [13]. FM results in a negative impact on their personal, professional, and daily lives, besides causing impairment in their physical, psychological, and social functioning [19]. To our knowledge, this is the first study that evaluates the prevalence of FM among medical students in Saudi Arabia and the Arab world.

In our study, the prevalence of FM in medical students was found to be 9.6%, which is higher than that among pooled general populations in the region (4.4%) [4]. This finding broadly supports the results of previous studies in the area linking FM with medical school-related stress. Omair et al. [20] have recently reported that the prevalence of FM among physicians in training in Riyadh is 8.2%, and they had used a similar questionnaire to the one used in this study. This result may indicate that the stress level continues to be significantly elevated during the undergraduate study period. It also appears to remain high during the internship, postgraduate training, and subsequently extends to the physician's personal, everyday life [21,22]. Therefore, medical students and professionals are inevitably at risk of developing FM at some point in their lives.

The findings related to the levels of FM in this study are higher compared to a study carried out by Eyigor et al., which targeted medical students and showed drastically different results with a prevalence of 2.0% [23]. This discrepancy could be attributed to the fact that they had applied the ACR 1990 diagnostic criteria in their study, which ultimately depended on the history of chronic pain and tender point examination, which led to concerns being raised on their reliability and validity and eventually resulted in the recommendation that their use in the clinical practice is stopped [24]. ACR 1990 was eventually replaced with ACR 2010 diagnostic criteria, which eliminated multiple problems associated with prior approaches. One among them was the variability of the amount of force exerted by physicians at the time of the physical examination, which had resulted in a more subjective impression and diagnosis [18].

Another interesting finding of our study was the higher prevalence of FM among students in the earlier, basic science years at the medical school when compared to those in the clinical-based training years. Our initial hypothesis had been that FM was highly related to the stress medical schools put their students under. Hence, we had expected the amount of stress to be significantly high in the first two years and that would continue to increase in the subsequent years of the training. However, our findings were inconsistent with this hypothesis. Many previous studies conducted in North America have reported that during the academic progression of students through medical school, their mental health worsens over the years [25,26]; our rather contradictory result may be due to students' ability to find and develop methods to cope with stress as they advance in their academic career. In addition, another study has found that there is usually a lower incidence of failure in later years of medical training, making students more self-assured and less stressed, as reported by Abdulghani et al. [13]. But the prevalence has been reported to pick up in the final year of medical school, and this finding supports our theory; this is attributed to students starting to experience new worries regarding the impending internship year and future residency.

Surprisingly, we found that more than three-quarters (77.8%) of the students with a physician's diagnosis of FM did not fulfill the FM diagnostic criteria, which is consistent with a previous study by Walitt et al. [27], which reported that three-quarters of the United States population who reported a clinical diagnosis of FM did not satisfy the diagnostic criteria. Many studies have been exploring this discordance between the FM diagnosis and the diagnostic criteria. A recent study conducted by Srinivasan et al. [28] investigated this concordance issue. Thus, several researchers have also started to raise the following question: "Why are patients who do not fulfill the criteria diagnosed clinically with fibromyalgia?"

A considerable number of FM cases do not exactly follow a standardized set of symptoms or fulfill the diagnostic criteria; however, it is not considered to be a diagnosis of exclusion, although some physicians have classified it as such since they often establish this diagnosis after negative testing for other differentials. Another possible explanation for this discordance is that FM symptoms' severity typically waxes and wanes throughout the disease course [29]. Hence, in our case, the participants who did not satisfy the criteria may not have had significantly severe symptoms at the time they completed the study questionnaire. Another possible explanation is that physicians usually do not follow and appreciate the FM criteria, but they still need to label serious, troublesome, but borderline FM symptoms and manage them accordingly.

Another important finding of the present study is that a significant number of participants (with and without FM) reported sleep problems. Our results showed that medical students overall, across different academic levels, are experiencing sleeping problems in similar proportions. Although sleep disturbance is one of the recognized FM symptoms, we did not assess this in an objective method. However, it was frequent among our participants, and many reported that the number of hours they had slept in the past two weeks was less than what is recommended by the American Academy of Sleep Medicine and the Sleep Research Society for a healthy adult, which is more than seven hours [30].

It is well established that FM can adversely affect the quality of life [19]. We believe that FM could affect the level of performance and productivity of medical students. However, a previous study by Abdulghani et al. [13] showed that there was neither an effect of stress on the academic grade nor an increase in absenteeism among the medical students. Nevertheless, it showed that stress was remarkably related to the students' physical problems. Although our data were insufficient to support this theory, we recommend that a future study be conducted to evaluate this aspect, which is important to all medical students.

One limitation of this study was its cross-sectional study design. It was based on self-reported information provided by students. Accordingly, there is some potential for reporting bias, which may have been due to students' perception of the questions. Furthermore, due to the study design, it was unfeasible to evaluate the risk factors for the development of FM among medical students. In spite of these limitations, we believe our study carries significant importance as it adds to the limited literature on FM among medical students; we also believe it will enable and encourage other researchers to further ascertain and study various possible predictors and characteristics of FM among medical students and build upon them to reach a more comprehensive understanding of the issue.

## Conclusions

Medical students carry a greater risk for developing FM compared to the general population as per the high prevalence of FM among them shown in this study, which used the 2010 ACR FM criteria. Multiple factors contribute to the development of FM, and stress is one of the most significant among these factors, especially among medical students as they tend to experience it on a daily basis. This issue cannot be neglected and should be addressed seriously, as it could affect their overall performance and eventually their medical practice in the future.
